# Comparative Transcriptomics Analysis of the Responses of the Filamentous Fungus *Glarea lozoyensis* to Different Carbon Sources

**DOI:** 10.3389/fmicb.2020.00190

**Published:** 2020-02-18

**Authors:** Ke Zhang, Baoqi Huang, Kai Yuan, Xiaojun Ji, Ping Song, Qingqing Ding, Yuwen Wang

**Affiliations:** ^1^College of Biotechnology and Pharmaceutical Engineering, Nanjing Tech University, Nanjing, China; ^2^Department of Geriatric Oncology, The First Affiliated Hospital of Nanjing Medical University, Nanjing, China; ^3^School of Food Science and Pharmaceutical Engineering, Nanjing Normal University, Nanjing, China

**Keywords:** transcriptome, pneumocandin B_0_, fructose, lipids, *Glarea lozoyensis*

## Abstract

The natural product pneumocandin B_0_ is the precursor of the antifungal drug caspofungin. We found that replacing glucose in the initial fermentation medium with 20 g/L fructose is more conducive to pneumocandin B_0_ production and biomass accumulation. In order to explore the mechanism of the different metabolic responses to fructose and glucose, we used each as the sole carbon source, and the results showed that fructose increased the total pneumocandin B_0_ yield and biomass by 54.76 and 13.71%, respectively. Furthermore, we analyzed the differences of gene expression and metabolic pathways between the two different carbon sources by transcriptomic analysis. When fructose was used as the carbon source, genes related to the pentose phosphate pathway (PPP), glycolysis and branched-chain amino acid metabolism were significantly upregulated, resulting in increased intracellular pools of NADPH and acetyl-CoA in *Glarea lozoyensis* for cell growth and pneumocandin B_0_ product synthesis. Interestingly, the pneumocandin B_0_ biosynthetic gene cluster and the genes of the TCA cycle were significantly downregulated, while the FAS genes were significantly upregulated, indicating that more acetyl-CoA was used for fatty acid synthesis. In particular, we found that excessive synthesis of fatty acids caused lipid accumulation, and lipid droplets can sequester lipophilic secondary metabolites such as pneumocandin B_0_ to reduce cell damage, which may also be an important reason for the observed increase of pneumocandin B_0_ yield. These results provide new insights into the relationship between pneumocandin B_0_ biosynthesis and carbon sources in *G. lozoyensis*. At the same time, this study provides important genomic information for improving pneumocandin B_0_ production through metabolic engineering strategies in the future.

## Introduction

In nature, fungi are challenged by a variety of biotic and abiotic stressors, ranging from attack by other microorganisms to nutrient deficiencies or extremes of pH and temperature. As a physiological reaction, they produce a large number of secondary metabolites (SMs), including antibiotics, cytochromes, and other active substances, some of which are applied as antitumor of cholesterol-lowering drugs. (Ruiz et al., 2010; Macheleidt et al., 2016). Among them, antibiotics are arguably the most important secondary metabolites, and are also the focus of a large body of research (Li and Tan, 2017). Pneumocandin B_0_ (PB_0_), the precursor of the antifungal drug caspofungin, is a secondary metabolite of the fungus *G. lozoyensis*. Due to its mild side effects, low drug resistance and low toxicity, it has become a focus of the search for new antibiotics (Bills et al., 1999; Connors and Pollard, 2004; Youssar et al., 2012).

During the culture process, nutrient components and metabolic compounds in the medium have a great influence on the synthesis of SMs (Sanchez and Demain, 2002). Among them, the carbon source is the most important influencing factor, and optimization of carbon sources has been the subject of continuous research (Kayali et al., 2011). Tkacz et al. (1993) found that using 100 g/L mannitol as the sole carbon source improved the yield of PB_0_ to 800 mg/L. Connors et al. (2000) found that by using a high concentration of 125 g/L fructose as the sole carbon source, the fermentation time of PB_0_ can be effectively reduced to 375 h, while also reducing the concentration of by-products and obtaining a final PB_0_ yield of approximately 400 mg/L. In our previous study (Song et al., 2018b), 20 g/L glucose and 80 g/L mannitol were used as carbon source, and an improved PB_0_ yield of 2768 mg/L was obtained.

Glucose is often used as a fast-acting carbon source, because it is believed to produce higher biomass growth than other carbohydrates (Wu et al., 2005). However, it was also reported that glucose inhibits the production of secondary metabolites (Demain, 1989). As a structural isomer of glucose, fructose bypasses the rate-limiting enzyme 6-phosphate fructokinase and enters the glycolysis pathway, which is more efficient than glucose metabolism (Fraenkel, 1968; Aristidou et al., 2010). Many studies have also confirmed that fructose is more suitable as a carbon source for microbial fermentation than glucose. Aristidou et al. (2010) suggested that fructose reduces the production of acetic acid compared to glucose, increasing the biomass of *Escherichia coli* by 40%. Li et al. (2015) found that when *Actinosynnema mirum* was cultured with fructose as the sole carbon source, the yield of Ansamitocin P-3 was increased fourfold compared to glucose. These results indicated that fructose could replace glucose as a carbon source for *G. lozoyensis* fermentation, but the effects of fructose on the metabolism of *G. lozoyensis* remain to be elucidated.

As technology advances, various “omics” techniques can help us fully understand the molecular mechanisms that respond to changes of environmental conditions (Bi et al., 2018). For example, Chen et al. (2013) clarified the mechanism of PB_0_ biosynthesis using genomics, while Song et al. (2018b) used metabolomics to determine that acetyl-CoA and NADPH are major factors limiting PB_0_ biosynthesis. RNA sequencing (RNA-Seq) techniques can provide massive amounts of sequence data for analysis of gene expression, which can be used to construct a complete view of differentially expressed genes and clarify the function of the corresponding metabolic pathways by comparing global transcriptomic changes (Oshlack et al., 2010; Wang et al., 2010; Ren et al., 2017).

In this study, in order to better understand the mechanism of action of different carbon sources in *G. lozoyensis*, we performed a comparative transcriptome analysis of *G. lozoyensis* cells grown with either fructose or glucose as sole carbon source. The results showed that fructose as a carbon source is more conducive to the production of NADPH and acetyl-CoA, as well as the accumulation of lipids. This study deepens our understanding of the mechanisms by which carbon sources influence the metabolism of *G. lozoyensis* and provides a theoretical basis for further genetic engineering of this promising antibiotic producer.

## Materials and Methods

### Strain and Culture Conditions

*Glarea lozoyensis* (CCTCC M 2019020) was preserved in the China Center for Type Culture Collection (Qin et al., 2016).

*G. lozoyensis* was seeded into 250 mL Erlenmeyer flasks containing 50 mL of seed medium and cultivated at 25°C and 220 rpm for 5 days. A sample comprising 5 mL of the resulting seed culture (v_0_) was centrifuged at 5000 g for 10 min, and the supernatant (v_1_) collected to determine the packed mycelial volume, defined as PMV = (v_0_−v_1_)/v_0_ × 100%. Then, the PMV was adjusted to 35%, and 10% (v/v) of the adjusted seed culture with a PMV of 35% was used to inoculate 50 mL of fermentation medium, which was cultured at 25°C and 220 rpm for 21 days. The seed medium contained (per liter): glucose 40 g, soybean powder 20 g cotton seed powder (Beijing Hongrunbaoshun Technology Co., Ltd., China) 10 g, corn steep liquor 10 g, KH_2_PO_4_ 1.5 g and trace elements solution (Connors et al., 2000) 10 mL. The trace elements solution contained (per liter): FeSO_4_⋅7H_2_O 1.0 g; MnSO_4_^.^H2O 1.0 g; ZnSO4^.^7H_2_O 1 g, CuCl_2_^.^2H_2_O 0.025 g; CaCl_2_^.^2H_2_O 0.1 g; H_3_BO_3_ 0.056 g; (NH_4_)Mo_7_O_24_⋅4H_2_O 0.019 g; and 12 N HCl 50 mL. The initial pH was adjusted to 5.0. The initial fermentation medium consisted of (per liter): glucose 20 g, mannitol 80 g, cotton seed power 28 g, corn gluten meal (Shandong Runyin Biological Chemical Co., Ltd., China) 10 g, K_2_HPO_4_ 2.5 g, Fe_3_(PO4)_2_ 0.5 g, and MnSO_4_.**H**_2_O 0.3 g.

### RNA Extraction, Library Construction, and RNA-Seq

For the analysis of the transcriptome, *G. lozoyensis* was cultured for 18 days with either glucose or fructose as carbon sources, after which the mycelia were collected, frozen immediately in liquid nitrogen and stored at −80°C until RNA extraction. Total RNA of each sample was extracted according to the instructions manual of the TRIzol Reagent (Life Technologies, United States). RNA integrity and concentration were checked using an Agilent 2100 Bioanalyzer (Agilent Technologies, Inc., Santa Clara, CA, United States). The mRNA was isolated using a NEBNext Poly(A) mRNA Magnetic Isolation Module (E7490; New England BioLabs, United States). The cDNA library was constructed following the manufacturer’s instructions of the NEBNext Ultra RNA Library Prep Kit for Illumina (E7530, NEB) and NEBNext Multiplex Oligos for Illumina (E7500, NEB). Briefly, the enriched mRNA was fragmented into approximately 250∼300 bp RNA inserts, which were used to synthesize the first- and second-strand cDNA. The double-stranded cDNA was subjected to end-repair/dA-tail and adaptor ligation. The suitable fragments were isolated using Agencourt AMPure XP beads (Beckman Coulter, Inc., United States), and enriched by PCR amplification. Finally, the constructed cDNA libraries were sequenced on an Illumina HiSeq2000 sequencing platform.

### Transcriptome Analysis Using Reference Genome-Based Reads Mapping

Low quality reads, such as adaptor, unknown nucleotides > 5%, or Q20 < 20% (percentage of sequences with sequencing error rates < 1%), were removed using a Perl script. The clean reads that were filtered from the raw reads were mapped to the *G. lozoyensis* genome using Tophat2 software (Kim et al., 2013). The aligned records in BAM/SAM format were further examined to remove potential duplicate molecules. Gene expression levels were estimated using FPKM values (fragments per kilobase of exon per million fragments mapped) using Cufflinks software (Trapnell et al., 2010).

### Identification of Differentially Expressed Genes

DESeq (Anders and Huber, 2010) and *Q*-value were employed to evaluate differential gene expression between *G. lozoyensis* mycelia grown on glucose and fructose, respectively. Differences of gene abundance between two samples were calculated based on the relative ratios of the FPKM values (FPKM > 1). The false discovery rate (FDR) control method was used to identify the threshold of the *P*-value in multiple tests in order to compute the significance of the differences. Here, only genes with an absolute value of the log2 fold change ≥1 and FDR significance score <0.05 were used for subsequent analysis.

### Analysis of Biomass, PB_0_, and Total Lipids

The biomass concentration and yield of PB_0_ were determined using the methods described in our previous study (Song et al., 2018b). The PB_0_ yield in the intracellular and extracellular fractions was determined by centrifuging 1 mL of the fermentation broth at 3000 g for 10 min. The supernatant was diluted 5 times with ethanol and the PB_0_ content measured by HPLC. The yield of PB_0_ in the intracellular fraction was calculated by subtracting the extracellular PB_0_ concentration in the supernatant from the concentration in the fermentation broth. To measure the biomass accumulation (dry cell weight, DCW), a sample comprising 5 mL of the *G. lozoyensis* fermentation broth was centrifuged for 10 min at 8000 g, washed 3 times with distilled water, and dried at 80°C until a constant weight. For metabolite extraction, a sample comprising 1 mL of the *G. lozoyensis* fermentation broth was mixed with 4 mL ethyl alcohol and shaken on an electronic oscillator for 10 min. The extract was then centrifuged for 5 min at 8000 g, and the supernatant was analyzed on a Dionex HPLC system (Dionex P680 pump, Chromeleon controller, and Dionex UVD 170U Detector; Dionex Corporation, United States). The yield of total lipids was determined the same way as published in our previous study (Qu et al., 2011).

### Validation of Expression Values Using Quantitative Real-Time PCR (qRT-PCR)

To verify the transcriptomic data, the same samples used for RNA-seq were also analyzed by qRT-PCR, as described previously (Ren et al., 2017). The qRT-PCR primers are listed in Supplementary Table S1.

## Results

### Effects of Different Carbon Source Combinations on the PB_0_ Yield and Biomass of *G. lozoyensis*

**FIGURE 1:**
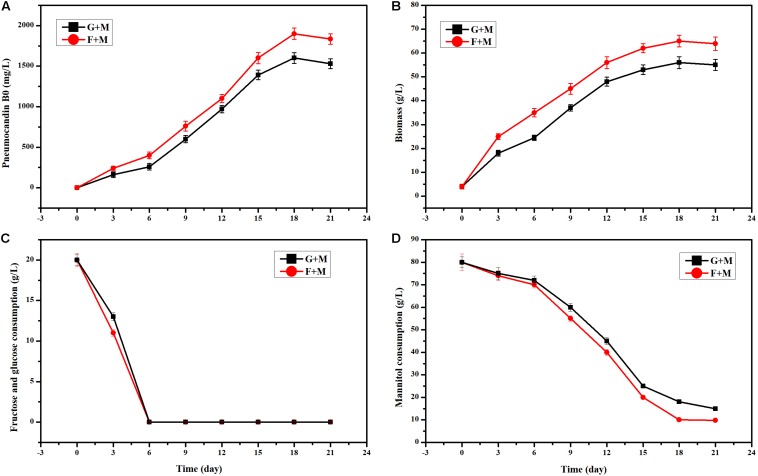
Effects of different carbon source combinations on PB_0_ production and biomass of *G. lozoyensis.* **(A)** Pneumocandin B_0_ (mg/L), **(B)** Biomass (g/L). **(C)** Fructose and glucose consumption, **(D)** Mannitol consumption, G + M: 20 g/L glucose and 80 g/L mannitol, F + M: 20 g/L fructose, and 80 g/L mannitol. The data were presented as the averages of three parallel samples, and the error bars indicate the standard deviation (SD) from the means of triplicates.

In the initial fermentation medium, 20 g/L glucose and 80 g/L mannitol were used as the carbon source. When we used 20 g/L fructose instead of 20 g/L glucose, the PB_0_ yield and biomass showed an obvious increase (Figures 1A,B). Especially at 6 days of fermentation, the change of PB_0_ yield and biomass was the most significant, with increases of 53.75 and 42.85%, respectively. This indicated that fructose as a carbon source is more conducive to the production of PB_0_ and the accumulation of biomass than glucose. When both glucose and fructose were depleted, *G. lozoyensis* started rapidly consuming mannitol (Figures 1C,D), demonstrating that *G. lozoyensis* preferred glucose and fructose to mannitol in the early stages of fermentation. Therefore, it was worthwhile to explore the different effects of fructose and glucose on PB_0_ production.

### Effects of Glucose and Fructose as Sole Carbon Source on the PB_0_ Yield and Biomass of *G. lozoyensis*

**FIGURE 2:**
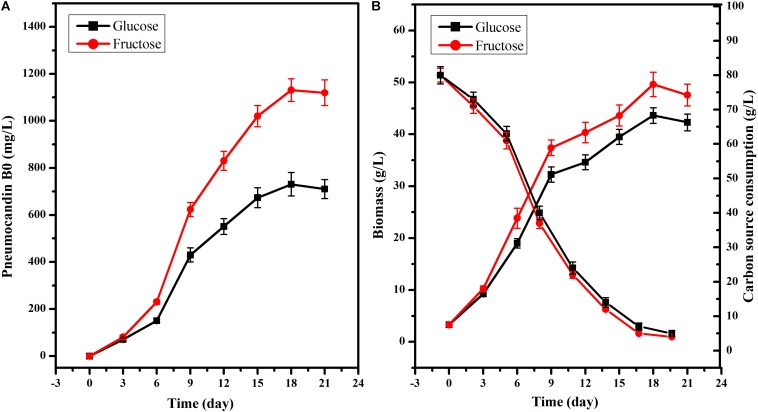
Effects of glucose and fructose on cell growth and PB_0_ production. **(A)** Pneumocandin B_0_ (mg/L), **(B)** Biomass (g/L), Fructose, and glucose consumption. The data were presented as the averages of three parallel samples, and the error bars indicate the standard deviation (SD) from the means of triplicates.

To further explore the different effects of fructose or glucose on PB_0_ production, 80 g/L glucose or fructose was used as the sole carbon source. Although the PB_0_ yield and biomass were different when glucose and fructose were used as substrate for culturing *G. lozoyensis* (Figure 2), the growth trend was consistent. There was no significant difference in the PB_0_ yield during the first 6 days of fermentation (165.60 ± 7.62 and 210.15 ± 9.25 mg/L at 6 days, respectively) (Figure 2A). After 6 days, PB_0_ production and biomass began to show significant differences and reached a maximum at 18 days. The yield of PB_0_ with fructose as carbon source was 54.76% higher than with glucose, at 1130.89 ± 48.49 and 730.76 ± 33.52 mg/L, respectively (Figure 2A). The biomass obtained with fructose increased by 13.71% compared to glucose, at 49.60 ± 2.35 and 43.62 ± 1.52 g/L, respectively (Figure 2B). In addition, the glucose and fructose consumption levels were practically the same throughout the fermentation process. Therefore, these results indicate that fructose may be considered a more efficient carbon source for the culture of *G. lozoyensis* than glucose.

### Effects of Two Carbon Sources on the Total Lipid Content, and the PB_0_ Yield in the Intracellular and Extracellular PB_0_ Fractions of the Fermentation Broth

**FIGURE 3:**
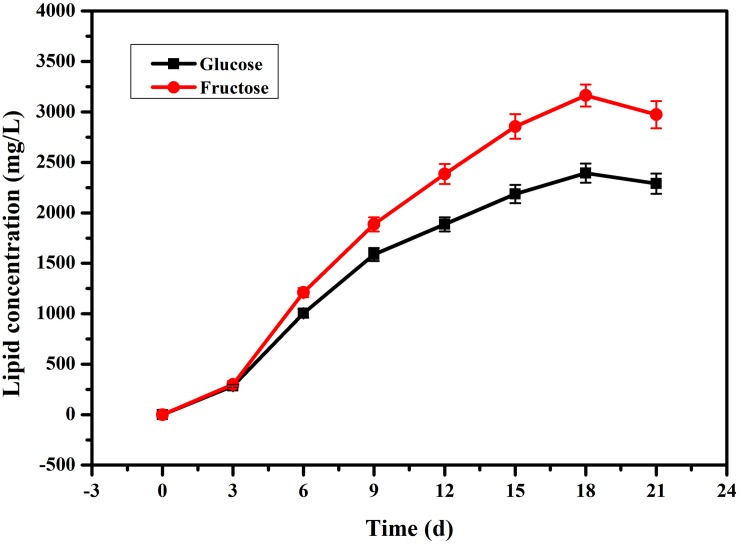
Comparison of the lipid concentrations in the mycelia of *G. lozoyensis* grown on glucose and fructose, respectively. The data were presented as the averages of three parallel samples, and the error bars indicate the standard deviation (SD) from the means of triplicates.

In our previous research (Song et al., 2018b), we found that fatty acids can affect PB_0_ biosynthesis. However, most of the fatty acid in the cell are present in the form of lipids and are not free (Sul and Wang, 1998; Cerón Garcí et al., 2000). Therefore, we examined changes in total lipid content and changes in intracellular and extracellular PB_0_ levels with the different carbon sources. As shown in Figure 3, we found that fructose as a carbon source was more conducive to lipid accumulation, which reached a maximum of 3162.86 ± 110.21 mg/L at 18 days, an increase of 33.85% compared to glucose (2362.86 ± 95.33 mg/L). At the same time, the growth trend of the total lipid content was consistent with the increase in PB_0_ production (Figure 2A), indicating that lipids are beneficial for PB_0_ production. In addition, when fructose was used as the carbon source, the proportion of intracellular PB_0_ at 18 days increased significantly compared with glucose, by 8.5% (Table 1). The results therefore indicate that fructose was a more favorable carbon source for the accumulation of lipids and intracellular PB_0_ than glucose.

**TABLE 1:** Intracellular and extracellular levels of PB_0_ and intracellular PB_0_ ratio with different carbon sources.

Time/d	Glucose				Fructose			
	Y_1_/mg/L	Y_2_/mg/L	Y_3_/mg/L	Y_4_/%	Y_1_/mg/L	Y_2_/mg/L	Y_3_/mg/L	Y_4_/%
6	322.04 ± 10.46	45.88 ± 1.47	276.16 ± 8.97	85.75 ± 2.57	397.24 1 ± 10.30	44.35 ± 1.15	352.89 ± 7.76	88.83 ± 1.87
18	965.26 ± 34.74	144.79 ± 5.21	820.47 ± 28.71	85.00 ± 2.21	1356.56 ± 36.63	88.17 ± 2.38	1268.39 ± 34.24	93.50 ± 1.68

Y_1_, total PB_0_ yield; Y_2_, extracellular PB_0_ yield; Y_3_, Intracellular PB_0_ yield; Y_4_, intracellular PB_0_ yield ratio. Y_3_ = Y_1_-Y_2_; Y_4_ = (Y_1_-Y_2_)/Y_1_ * 100%.

### Illumina Sequencing and Functional Annotation

After observing the accumulation process of PB_0_ in *G. lozoyensis* cultured with two different carbon sources (Figure 2A), comparative transcriptomic analyses were performed at the pre-fermentation stage (6 days) during which the product began to accumulate, and the later-fermentation stage (18 days) during which the product accumulated to the maximum level.

To identify functional genes responsible for the changes in biomass and PB_0_ production under different carbon sources, total RNA was isolated from *G. lozoyensis* cultured for 6 and 18 days and subjected to Illumina sequencing. After data cleanup and quality checks, a total of 92.8 million 150 bp paired-end (PE) reads were obtained from the 8 fq files accounting for 62.7 Gb of raw data. The Q20 (percentage of bases with quality >20 in clean reads), Q30, and GC percentages for the clean datasets were 97.4/96.3%, 93.1/90.6%, and 49.7/49.7% (Supplementary Table S2). Using Tophat2 (Trapnell et al., 2009) and bowtie2 (Langmead, 2010) to compare the clean reads of each sample with the reference genome sequence of *G. lozoyensis*, we obtained 2.9 million high quality unique mapped reads (Supplementary Table S3). After assembly, a total of 13,692 transcripts were generated with a total length of 28,324,824 bp. Using RSEM (Dewey and Bo, 2011), the alignment results of bowtie2 were compared with transcripts and converted into FPKM values to obtain the expression levels of genes, i.e., the relative abundance of transcripts (Supplementary Figure S1). The raw sequencing data was stored in the NCBI Sequence Read Archive (SRA) database^1^ under the accession number SRP220084.

**FIGURE 4:**
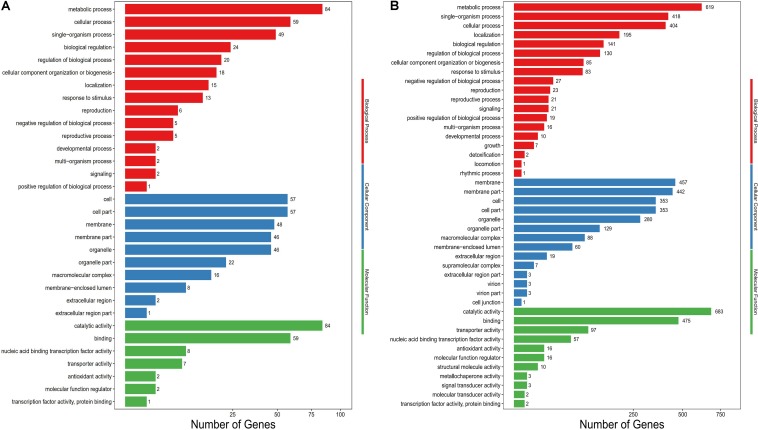
Gene ontology (GO) functional enrichment analysis of the differentially expressed genes (DEG). The X-axis represents the number of DEG, and the Y-axis lists GO terms. **(A)** G6 vs. F6; **(B)** G18 vs. F18. G6,G18: *G. lozoyensis* grown with glucose as carbon source taken at 6 and 18 days; F6,F18: *G. lozoyensis* grown with fructose as carbon source taken at 6 and 18 days.

For a better understanding of gene function in cellular processes, all unigenes were searched against the National Center for Biotechnology Information (NCBI) non-redundant protein (Nr) database using BLASTX with a cut-off *E*-value of 10^–5^ (Conesa et al., 2005). In order to obtain the gene ontology (GO) annotation information, the Nr BLAST results were imported into the Blast2 GO program and GO annotations for the unigenes were obtained (Götz et al., 2008). Based on the Blast2 GO comparison results, we performed GO term enrichment analysis with FDR ≤0.05 as the cutoff. All GO annotations of the unigenes were divided into the three functional categories biological process, cellular component and molecular function. However, the number of genes enriched in different GO terms in different fermentation stages (at 6 and 18 days) was also different (Figure 4). In the early stages of fermentation (at 6 days), there were no great differences in PB_0_ production with either glucose or fructose. Therefore, the number of genes enriched in GO terms was relatively small (771 unigenes), including the categories biological process (305; 39.56%), cellular component (303; 39.30%) and molecular function (163; 21.41%). In the late stage of fermentation (at 18 days), when *G. lozoyensis* was cultured with fructose as the carbon source, the number of genes enriched in GO terms increased significantly (5785 unigenes), including the categories biological process (2223; 38.43%), cellular component (2198; 37.99%) and molecular function (1364; 23.58%). In detail, the GO terms metabolic process (84; 619), membrane (48; 457), membrane part (46; 442), catalytic activity (84; 683), and binding (59; 475) accounted for a large proportion of the total, followed by the terms signal-organism process (49; 418), cellular process (59; 404), cell (57; 353), and cell part (57; 353). These results not only showed the specific distribution of differentially expression genes (DEGs) in the Gene Ontology classification, but also provided information on the regulation of a large number of genes.

By comparing the transcription levels of mycelia grown on different carbon sources, we found significant differences at 6 and 18 days. When *G. lozoyensis* was cultured with fructose as the carbon source, there were a total of 198 differentially expressed genes (DEGs; 123 up- and 75 downregulated) at 6 days (fold change ≥2 and false discovery rate ≤0.05), while there were a total of 1722 DEGs (874 up- and 848 downregulated) at 18 days. In order to understand their functions, these DEGs were all mapped to the related pathways in the Kyoto Encyclopedia of Genes and Genomes (KEGG) database and the most important biochemical metabolic pathways and signal transduction pathways were identified by significant KEGG pathway enrichment. After KEGG pathway enrichment analysis (Supplementary Figure S2), a total of 101 unigenes were identified as DEGs between G6 and F6, involved in processes including carbohydrate metabolism (12; 11.88%), amino acid metabolism (9; 8.91%), cell growth and death (4; 3.96%), signal transduction (9; 8.91%), and biosynthesis of other secondary metabolites (2; 1.98%). In addition, 555 unigenes were identified as DEGs between G18 and F18, involved in processes including carbohydrate metabolism (71; 12.79%), amino acid metabolism (54; 9.81%), cell growth and death (13; 2.34%), signal transduction (29; 5.23%), and biosynthesis of other secondary metabolites (12; 2.16%). The observed differences in the number of genes were consistent with the fermentation results.

### Differential Expression of Genes Involved in Central Carbon Metabolism

GO and KEGG enrichment analysis showed that genes involved in cell metabolism, and especially carbon metabolism, were significantly affected by the different carbon sources. Therefore, the focus of this study was the carbon metabolism of *G. lozoyensis*. Although glucose and fructose are isomers, they are metabolized differently by living organisms. Based on the obtained transcriptome information, carbohydrate metabolism pathways including glycolysis, the pentose phosphate pathway (PPP), the tricarboxylic acid (TCA) cycle, fructose and mannose metabolism, were studied. Compared with glucose, when *G. lozoyensis* was cultured with fructose as the carbon source, the ketohexokinase (KHX), which catalyzes the formation of fructose 1-phosphate, was upregulated 5.6-fold at 18 days. In addition, glucose-6-phosphate isomerase (GPI; upregulated 4.0-fold), 6-phosphofructokinase (PFK; upregulated 2.6-fold), fructose-bisphosphate aldolase (ALDO; upregulated 3.1-fold), glucose-6-phosphate dehydrogenase (G6PD; upregulated 2.4-fold), 6-phosphogluconate dehydrogenase (PGD; upregulated 6.4-fold), which are involved in glycolysis and the PPP, were significantly upregulated at 18 days. Among the genes related to the TCA cycle, pyruvate dehydrogenase (PDH), and ATP citrate lyase (ACL) were upregulated 2.5- and 4.4-fold at 18 days, while citrate synthase (CS) and succinate dehydrogenase (SDH) were downregulated 4.1- and 2.6-fold at 18 days. These results indicate that when fructose is used as a carbon source, it promotes glycolysis and the PPP, while inhibiting the TCA cycle compared with glucose.

### Differential Expression of Genes Involved in Amino Acid Metabolism

Pathways involved in amino acid metabolism are essential for the survival of all living organisms. Amino acid metabolism not only provides energy and intermediate metabolites for cell metabolism, but also plays an important role in signal transduction, regulation of gene expression, and antioxidant activity (Miflin and Lea, 1977; Wu, 2009; Wendisch, 2014). According to transcriptome analysis, when fructose was used as the carbon source, genes involved in the glycine, serine and threonine metabolism pathway, as well as the cysteine and methionine metabolism pathway were significantly upregulated at 6 and 18 days. These include L-serine/L-threonine ammonia-lyase (SDS), cystathionine gamma-lyase (CTH), cystathionine beta-synthase (CBS), L-cysteine desulfhydrase (CD), and cysteine-S-conjugate beta-lyase (metC). At the same time, genes involved in the metabolism of tryptophan, phenylalanine and tyrosine were also upregulated at 18 days, including indoleamine 2,3-dioxygenase (IDO), kynurenine 3-monooxygenase (KMO), kynureninase (kynU), 3-hydroxyanthranilate 3,4-dioxygenase (HAAO), acetyl-CoA C-acetyltransferase (atoB), L-tryptophan decarboxylase (TDC), tyrosine aminotransferase (TAT), 4-hydroxyphenylpyruvate dioxygenase (HPD), and homogentisate 1,2-dioxygenase (hmgA). These results indicate that when fructose was used ascholine dehydrogenase the carbon source, the metabolism of the above-mentioned amino acids was significantly enhanced, which provided more pyruvate and acetoacetyl-CoA for acetyl-CoA synthesis (Harris et al., 2005; Blachier et al., 2013).

### Differential Expression of Genes Involved in Fatty Acid Biosynthesis and the Pneumocandin Biosynthesis Pathway

PB_0_ is a lipopeptide composed of a hexapeptide and a fatty acid (Mizuno et al., 1977; Wichmann et al., 1989). Chen et al. (2013) speculated that PB_0_ is synthesized by a hybrid pathway comprising a polyketide synthase and non-ribosomal peptide synthetase (PKS-NRPS) by analyzing the structure and function of the gene cluster. To explore the metabolic mechanism of PB_0_ production, we analyzed the differentially expressed genes in the PKS-NRPS system and the fatty acid synthesis pathway (FAS) under different carbon sources by comparing the corresponding transcriptomes. At 6 days of fermentation, the expression of key genes in the fatty acid synthesis pathway was upregulated. These include acetyl-CoA carboxylase (ACC), β-ketoacyl-ACP reductase (FabG), and fatty acid synthase (FAS1), which are up-regulated by 3. 8-, 4. 5-, and 4.3-fold, respectively. At 18 days, the PB_0_ yield was significantly different, and the genes of the PKS-NRPS pathway (GLPKS4, GLNRPKS4, GL ligase, GLHYD, GLOXY1, GLOXY2, GLP450-1, and GLP450-2) were significantly downregulated. In addition, the expression levels of genes involved in the fatty acid synthesis pathway were significantly upregulated in the culture with fructose as the carbon source compared to glucose. In particular, both FabG and FAS1 were upregulated 8.1-fold. These transcriptomic data indicate that different expression levels of genes in the fatty acid synthesis pathway may affect the synthesis of PB_0_ in *G. lozoyensis*.

### Verification of the Transcriptomic Data via qRT-PCR

To verify the results of transcriptome analysis, the transcription of the most relevant differentially expressed genes (PFK, G6PD, CS, CTH, FabG, GLPKS4, ACL, GL ligase) was analyzed using qRT-PCR (Supplementary Figure S3). The results of qRT-PCR conducted with the same RNA samples that were used for RNA-seq indicated that the gene expression levels were consistent with those inferred by transcriptome analysis, indicating that the RNA-seq data are reliable.

## Discussion

PB_0_, the precursor of the antifungal drug caspofungin, is a secondary metabolite of the fungus *G. lozoyensis* (Bills et al., 1999; Youssar et al., 2012), and its biosynthesis is affected by the types and concentrations of nutrients in the medium. Among them, the carbon source is a key factor affecting cell growth and PB_0_ production (Sanchez and Demain, 2002; Kayali et al., 2011). However, recent research on *G. lozoyensis* has mainly focused on biosynthetic gene clusters and fermentation regulation strategies (Chen et al., 2013, 2015; Song et al., 2018a, c), while the mechanisms that regulate the carbon source metabolism are still unknown. In this study, when using 80 g/L fructose as the sole carbon source instead of 80 g/L glucose (Figure 2), the biomass and PB_0_ yield at 18 days of fermentation were increased by 13.71 and 54.76%, respectively. In order to understand the metabolic mechanism by which carbon sources affect the PB_0_ yield, we compared the transcriptomes of *G. lozoyensis* mycelia grown with fructose and glucose as sole carbon source, respectively. All the genes mentioned in this paper are listed in Supplementary Table S4.

**FIGURE 5:**
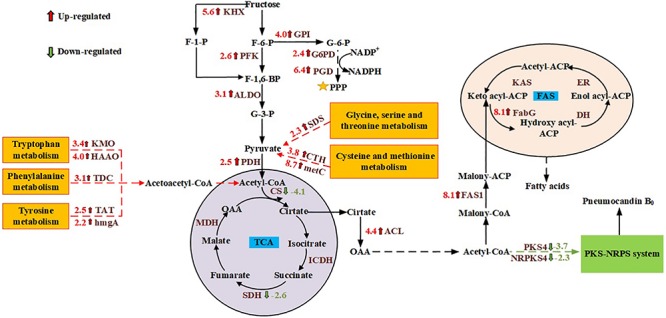
Changes in the transcript abundance of genes involved in central metabolic pathways, fatty acid biosynthesis, and the PKS-NRPS pathway. Key enzymes with logFC values are included in the map with their names written out. Red represents up- and blue represents downregulation. KHX, ketohexokinase; PFK, 6-phosphofructokinase; ALDO, fructose-bisphosphate aldolase; GPI, glucose-6-phosphate isomerase; G6PD, glucose-6-phosphate dehydrogenase; PGD, 6-phosphogluconate dehydrogenase; PDH, pyruvate dehydrogenase; CS, citrate synthase; ACL, ATP citrate lyase; ICDH, isocitrate dehydrogenase; SDH, succinate dehydrogenase; MDH, malate dehydrogenase; FAS1, fatty acid synthase; PKS4, polyketide synthase; NRPS4, non-ribosomal peptide-synthetase; FabG, β-ketoacyl-ACP reductase; DH, 3-hydroxyacyl-ACP dehydratase; ER, enoyl reductase; KAS, β-ketoacyl-ACP synthase; SDS, L-serine/L-threonine ammonia-lyase; CTH, cystathionine gamma-lyase; metC, cysteine-S-conjugate beta-lyase; KMO, kynurenine 3-monooxygenase; HAAO, 3-hydroxyanthranilate 3,4-dioxygenase; TDC,L-tryptophan decarboxylase; TAT, tyrosine aminotransferase; hmgA, homogentisate 1,2-dioxygenase.

It is well known that acetyl-CoA and NADPH are key precursors in cell metabolism and an important component of the cell’s energy supply (Pietrocola et al., 2015). In most microorganisms, the key enzymes G6PDH and PGD in the PPP are thought to be the main source of NADPH (Bi et al., 2018). NADPH can be used not only for PB_0_ biosynthesis, but also for cell growth and maintaining the redox balance (Wang et al., 2017). According to the transcriptomic data, G6PD and PGD were respectively upregulated 2.4- and 6.4-fold with fructose as the carbon source. Therefore, we speculated that fructose as a carbon source can upregulate the PPP to a certain extent to produce more NADPH for cell growth and metabolism. In addition to NADPH, acetyl-CoA is a central metabolic intermediate in various biochemical pathways and an indispensable precursor for fatty acid biosynthesis (Lian et al., 2014). Various catabolic reactions can produce acetyl-CoA to provide energy to cells, including glycolysis, beta-oxidation, and catabolism of branched-chain amino acids (Trotter, 2001; Vorapreeda et al., 2012). As mentioned in our previous work, acetyl-CoA for PB_0_ synthesis is mainly produced by ACL (Song et al., 2018a). In this study, we found that ACL expression levels at 18 days were significantly upregulated, approximately 4.4-fold, when fructose was used as the carbon source. In addition, in the metabolism of fructose and mannitol, KHK was upregulated 5.6-fold, indicating that KHK can convert fructose into fructose 1-phosphate and thereby channel it to the glycolysis pathway. Li et al. (2015) obtained similar results. In addition, the genes involved in the glycolysis pathway also exhibited significant changes. When fructose was used as the carbon source, the PFK responsible for catalyzing the formation of fructose 1,6-diphosphate from fructose-6-phosphate was upregulated 2.6-fold. The expression of other enzymes involved in the glycolysis pathway also increased, such as GPI and ALDO, which were upregulated 4.0- and 3.1-fold, respectively. At the same time, some amino acid metabolic pathways were also promoted when fructose was used as the carbon source. For example, the metabolism of glycine, serine and threonine, as well as the cysteine and methionine metabolism, tryptophan metabolism and tyrosine metabolism were all upregulated. Among them, serine, cysteine, tryptophan and tyrosine-related catabolic genes were upregulated the most. The increase of the expression of genes related to glycolysis and amino acid metabolism may cause pyruvate accumulation (Harris et al., 2005). In response to this change, the PDH responsible for catalyzing the formation of acetyl-CoA from pyruvate was significantly upregulated 2.5-fold. Therefore, the cells growing on fructose may have accumulated more acetyl-CoA, providing precursors for biosynthesis. Interestingly, the TCA cycle, which also uses acetyl-CoA, was inhibited. This indicates that more acetyl-CoA may have been used for the synthesis of PB_0_ and fatty acids (Figure 5).

Since the synthesis of both PB_0_ and fatty acids requires large amounts of acetyl-CoA and NADPH, we analyzed the differentially expressed genes in the PB_0_ synthetic gene cluster and the FAS pathway to explore the differences in the effects of carbon sources on the two products. Transcriptome analysis showed that when *G. lozoyensis* was cultured with fructose as the carbon source, the expression of genes in the PB_0_ synthetic gene cluster was downregulated, while the PB_0_ yield was increased by 54.76%. Such counterintuitive findings were rarely reported, but similar results were also obtained for penicillin production. Guedes and Leitão (2012) reported the stimulation of penicillin production by high salt concentrations, but the levels of penicillin biosynthetic genes were not significantly correlated with the penicillin yield. However, in contrast with the downregulation of the PB_0_ biosynthesis genes, the expression of FAS genes was significantly upregulated. In particular, FAS1 and FabG were upregulated 8.1-fold, which indicates that more acetyl-CoA was used for fatty acid synthesis. However, only a small amount of fatty acids is present in the free form, and most are in the form of lipids in the cells (Sul and Wang, 1998; Cerón Garcí et al., 2000). As common macromolecular lipophilic structures, lipid droplets and membranes can provide a storage compartment for lipophilic secondary metabolites such as intracellular polyketides and carotenoids, and avoid their potential toxic effects on the producing cells (Meadows et al., 2016; Tan and Liu, 2016). Ma et al. (2019) overexpressed key genes involved in fatty acid synthesis and fatty-acid glyceryl ester synthesis, thereby increasing the intracellular lipid accumulation, which increased lycopene production by 25% relative to the original strain. In addition, Larroude et al. (2018) found that high-yielding strains of β-carotene producers often display high lipid accumulation. Samuel (2011) proposed that fructose is beneficial for the accumulation of fatty acids and lipids. When fructose was used as the carbon source, the total lipid content at 18 days increased by 33.85% compared to glucose (3162.86 ± 110.21 and 2,362.86 ± 95.33 mg/L, respectively) and the proportion of intracellular PB_0_ increased by 8.5% (Figure 3 and Table 1). We therefore hypothesized that increased lipid accumulation may reduce damage to cells by secondary metabolites such as PB_0_, thereby increasing PB_0_ production.

## Conclusion

With fructose as the fermenting carbon source, the biomass and PB_0_ yield of *G. lozoyensis* respectively increased by 13.71 and 54.76%. We analyzed the differences of gene expression and metabolic pathways between mycelia grown on different carbon sources using transcriptomics. The results showed that when fructose was used as the carbon source, PPP, glycolysis and branched-chain amino acid metabolism of *G. lozoyensis* were promoted, which may have provided more NADPH and acetyl-CoA for biosynthesis. Furthermore, downregulation of the PB_0_ biosynthesis gene cluster and the TCA cycle resulted in more acetyl-CoA for fatty acid synthesis. In addition, our data also confirmed that an increase of fatty acid synthesis further leads to the accumulation of lipids, and the effect of lipid-mediated sequestration reduces the damage caused by secondary metabolites such as PB_0_ to the producing cells, thereby increasing the yield of PB_0_. These results provide new insights into the relationship between PB_0_ biosynthesis in *G. lozoyensis* and carbon sources. At the same time, this study provides important genomic information and new directions for improving PB_0_ yield by engineering FAS in the future.

## Data Availability Statement

The datasets generated for this study can be found in the NCBI, Sequence Read Archive, SRP220084, https://www.ncbi.nlm.nih.gov/sra.

## Author Contributions

KZ, BH, and KY carried out the experiments. KZ and BH analyzed the transcriptome data and wrote the manuscript. PS, QD, XJ, and YW provided the idea for the project and are accountable for all aspects of the work. All authors approved the final manuscript.

## Conflict of Interest

The authors declare that the research was conducted in the absence of any commercial or financial relationships that could be construed as a potential conflict of interest.

## Footnotes

1 https://www.ncbi.nlm.nih.gov/sra
