# Supplementary material for: Comparative Transcriptomics Analysis of the Responses of the Filamentous Fungus Glarea lozoyensis to Different Carbon Sources
Source: Front Microbiol. 2020 Feb 18;11:190. doi: 10.3389/fmicb.2020.00190 (PMC7040073; doi:10.3389/fmicb.2020.00190)
Supplement: Supplementary file 1 [file Data_Sheet_1.docx]

**Fig. S1** The Statistics on the number of genes in different expression levels with FPKM >1. G6,G18: *G. lozoyensis* grown with glucose as carbon source taken at 6 and 18 days; F6,F18: *G. lozoyensis* grown with fructose as carbon source taken at 6 and 18 days.


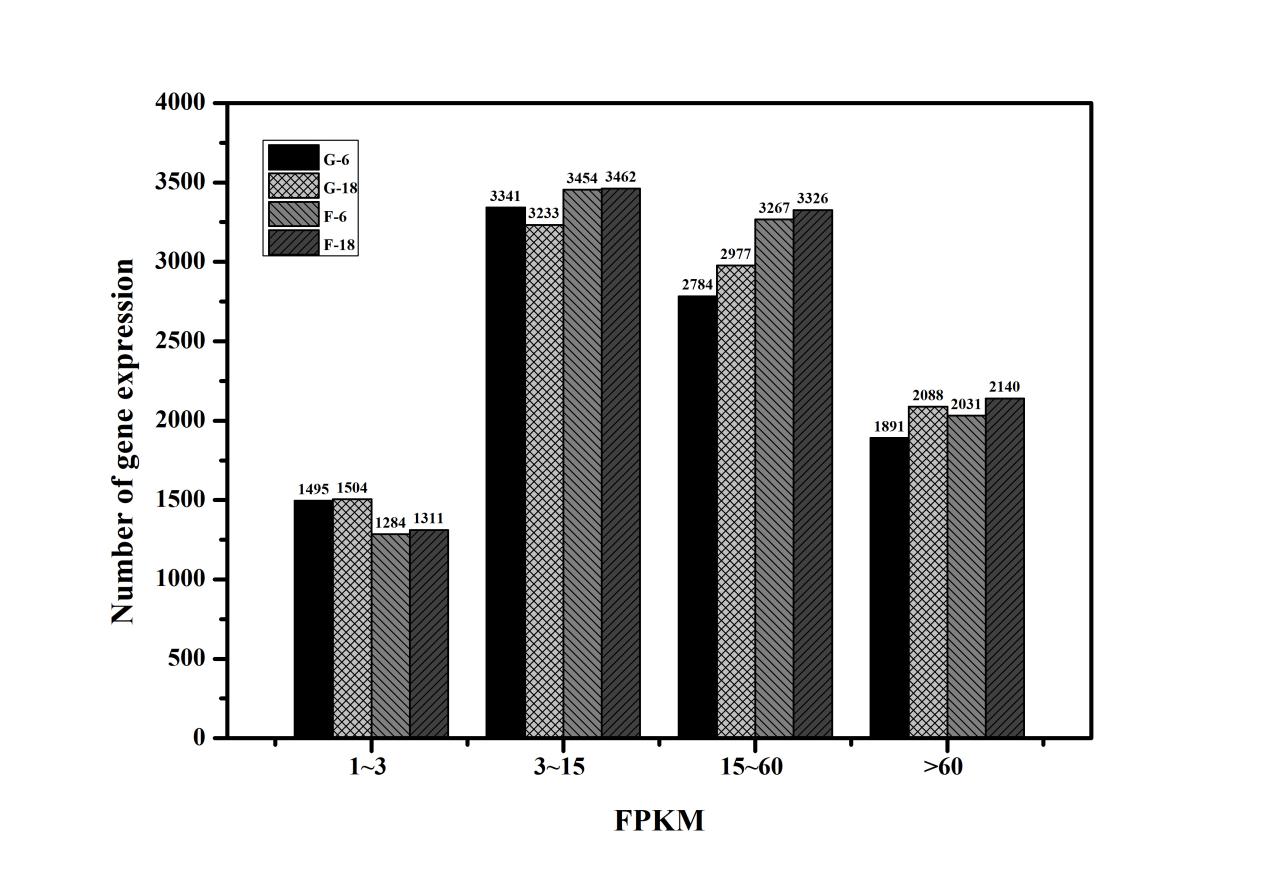


**Fig. S2** KEGG pathway enrichment analysis of the differentially expressed genes (DEG) with a FDR of ≦0.05.X axis represents number of DEG. Y axis represents the name of the KEGG metabolic pathway. (A) G6 VS F6; (B ) G18 VS F18. G6,G18: *G. lozoyensis* grown with glucose as carbon source taken at 6 and 18 days; F6,F18: *G. lozoyensis* grown with fructose as carbon source taken at 6 and 18 days.


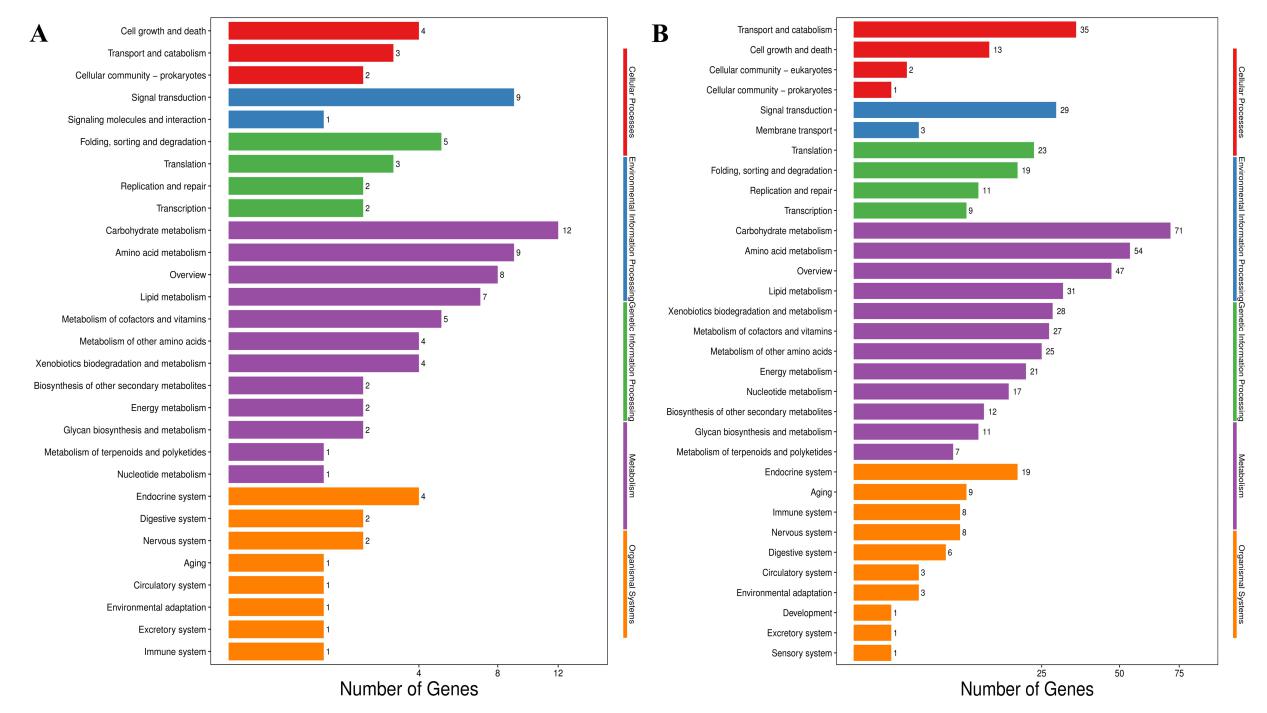


**Fig. S3** Validation of the gene expression profiles of the PFK, G6PD, CS, ACL, CTH, FabG, GLPKS4 and GL ligase genes by quantitative real-time PCR (qRT-PCR). The results were normalized to the 18S rRNA. G6,G18: *G. lozoyensis* grown with glucose as carbon source taken at 6 and 18 days; F6,F18: *G. lozoyensis* grown with fructose as carbon source taken at 6 and 18 days. The data were presented as the averages of three parallel samples, and the error bars indicate the standard deviation (SD) from the means of triplicates


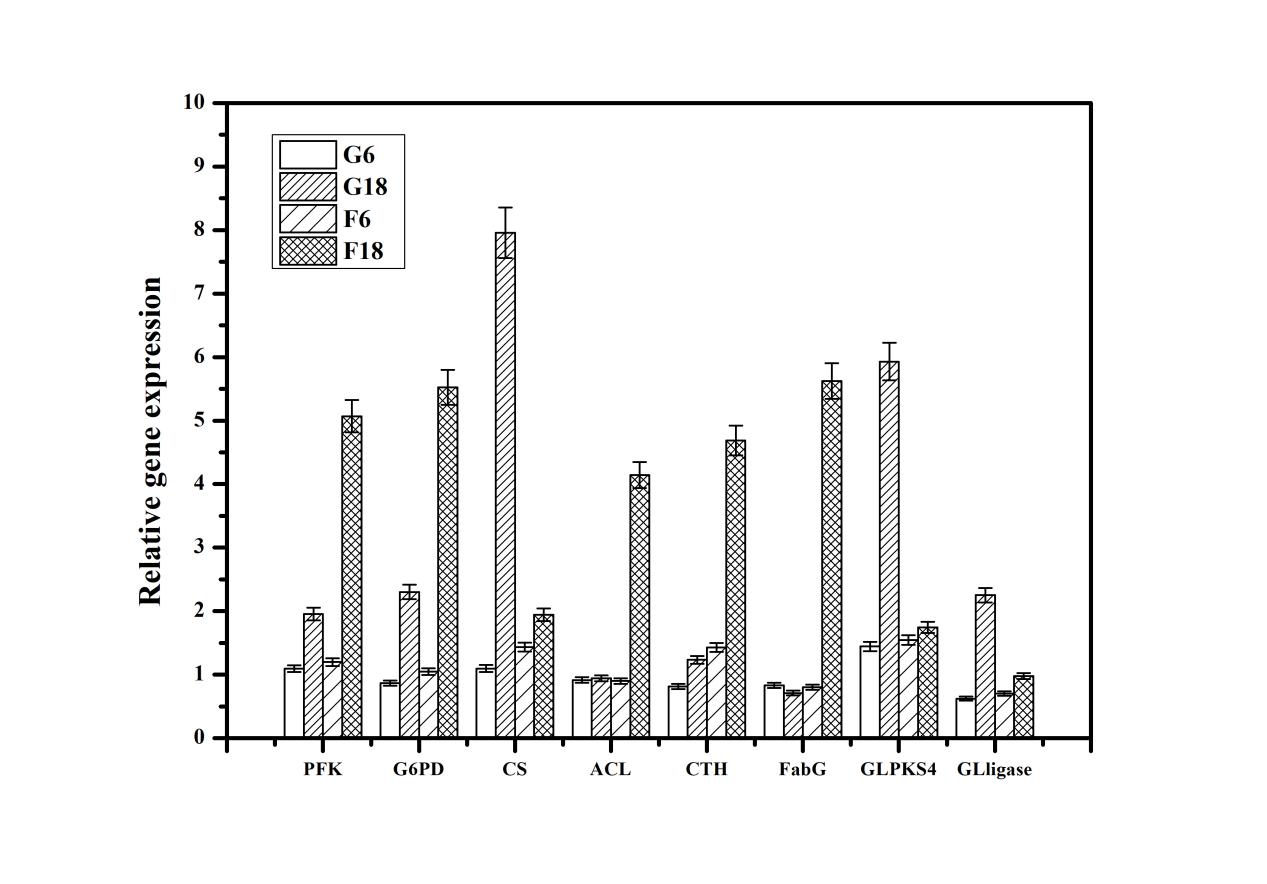


**Table S1** Primers used for quantitative real-time PCR (qRT-PCR).

| Gene name | Gene ID | Primer sequence (5-3’) |
| --- | --- | --- |
|  |  |  |
| G6PD | GLAREA_00837 | 1. ACCGTGATTGTCGTTCT   R-TCAGGTGCTGGGAGAC |
|  |  |  |
| PFK | GLAREA_02833 | F-GTGGCTGGCTTTCCG  R-GACCCAACTAGACCAACAATA |
|  |  |  |
| CS | GLAREA_01577 | 1. ACTATCATCCCTTCCTACA   R-GAACAGAACCTTCCCAT |
|  |  |  |
| CTH | GLAREA_05647 | 1. CCAGTGGGCATTTACG   R-GCCTTTGCGACTTGTG |
|  |  |  |
| FabG | GLAREA_10581 | F-ATGGCACCAGCAGC  R-CCACGACCAATACCG |
|  |  |  |
| GLPKS4 | GLAREA_10034 | 1. TCGTGGGTATGGGG   R-TGCGTTGCTGAGGAT |
|  |  |  |
| ACL | GLAREA_01672 | F-GCCGCTACACTGCTC  R-CATCTGCTGCCTTGAC |
|  |  |  |
| GL ligase | GLAREA_10043 | F-GATAAGGGACATCAGACAC  R-GAGCAGACAGCGACAA |
|  |  |  |
| 18S rRNA | endogenous control gene | F-CAGAGGACCCCAAACT  R-AGAGCCCAAGACCCA |
|  |  |  |

**Table S2** Summary of draft reads of samples by Illumina sequencing. The Q20 percentage is the proportion of nucleotides with quality values larger than or equal to 20; the Q30 percentage is the proportion of nucleotides with quality values larger than or equal to 30; the GC percentage is the proportion of guanidine and cytosine nucleotides among total nucleotides.

| Sample | Clean reads pairs | Clean bases | Length | Q20(%) | Q30(%) | GC(%) |
| --- | --- | --- | --- | --- | --- | --- |
| G6 | 22,548,440 | 6,764,532,000 | 150;150 | 97.4;96.2 | 93.1;90.5 | 49.9;49.9 |
| G18 | 26,003,945 | 7,801,183,500 | 150;150 | 97.4;96.5 | 93.1;91.0 | 49.6;49.5 |
| F6 | 20,752,119 | 6,225,635,700 | 150;150 | 97.4;96.4 | 93.1;90.8 | 49.8;49.8 |
| F18 | 23,508,815 | 7,052,644,500 | 150;150 | 97.3;96.1 | 93.0;90.2 | 49.4;49.4 |

**Table. S3** Reference gene comparison with total clean reads

| Sample | Total clean read pairs | Total mapped reads | Uniq mapped reads | Multiple mapped reads |
| --- | --- | --- | --- | --- |
| G6 | 22,548,440 | 18,638,772(82.66%) | 7,767,243(34.45%) | 10,871,529(48.21%) |
| G18 | 26,003,945 | 21,686,398(83.40%) | 7,991,125(30.73%) | 13,695,273(52.67%) |
| F6 | 20,752,119 | 17,202,526(82.90%) | 6,398,242(30.83%) | 10,804,284(52.06%) |
| F18 | 23,508,815 | 19,350,971(82.31%) | 6,894,133(29.33%) | 12,456,838(52.99%) |

**Table. S4** The gene list mentioned in this paper. G6,G18: *G. lozoyensis* grown with glucose as carbon source taken at 6 and 18 days; F6,F18: *G. lozoyensis* grown with fructose as carbon source taken at 6 and 18 days.

| Gene | Gene ID | Pathway | FPKM | | | |
| --- | --- | --- | --- | --- | --- | --- |
|  |  |  | G6 | F6 | G18 | F18 |
| GPI | GLAREA12579 | Glycolysis |  |  | 45.22 | 857.71 |
| PFK | GLAREA02833 | Glycolysis |  |  | 25.63 | 178.45 |
| ALDO | GLAREA11809 | Glycolysis |  |  | 121.28 | 1264.30 |
| G6PD | GLAREA00837 | PPP |  |  | 51.92 | 322.28 |
| PGD | GLAREA03380 | PPP |  |  | 80.12 | 1012.23 |
| PDH | GLAREA12506 | TCA |  |  | 85.72 | 463.10 |
| ACL | GLAREA01672 | TCA |  |  | 69.24 | 1687.28 |
| CS | GLAREA01577 | TCA |  |  | 749.50 | 51.35 |
| SDH | GLAREA04970 | TCA |  |  | 953.36 | 184.25 |
| KHX | XLOC001041 | Fructose and mannose metabolism |  |  | 40.51 | 2306.32 |
| SDS | GLAREA07335 | Glycine, serine and threonine metabolism | 35.21 | 125.37 | 69.51 | 280.45 |
| CBS | GLAREA07800 | Glycine, serine and threonine metabolism | 23.55 | 60.42 | 57.92 | 285.76 |
| CTH | GLAREA05647 | Cysteine and methionine metabolism | 20.56 | 81.73 | 149.02 | 2415.28 |
| CD | GLAREA00252 | Cysteine and methionine metabolism | 19.15 | 45.26 | 100.35 | 321.54 |
| metC | GLAREA04056 | Cysteine and methionine metabolism | 55.22 | 115.86 | 33.15 | 13786.20 |
| IDO | GLAREA03347 | Tryptophan metabolism |  |  | 25.44 | 333.47 |
| KMO | GLAREA04299 | Tryptophan metabolism |  |  | 28.54 | 407.50 |
| kynU | GLAREA11410 | Tryptophan metabolism |  |  | 90.15 | 290.56 |
| HAAO | GLAREA05397 | Tryptophan metabolism |  |  | 54.60 | 1023.91 |
| atoB | GLAREA04174 | Tryptophan metabolism |  |  | 41.77 | 493.59 |
| TDC | GLAREA08319 | Phenylalanine metabolism |  |  | 32.81 | 337.60 |
| TAT | GLAREA00731 | Tyrosine metabolism |  |  | 42.13 | 194.61 |
| HPD | GLAREA03832 | Tyrosine metabolism |  |  | 16.30 | 584.39 |
| hmgA | GLAREA00182 | Tyrosine metabolism |  |  | 73.28 | 348.04 |
| ACC | GLAREA00402 | Fatty acid synthesis | 60.23 | 838.93 |  |  |
| FabG | GLAREA10581 | Fatty acid synthesis | 32.11 | 726.57 | 66.32 | 18209.48 |
| FAS1 | GLAREA08410 | Fatty acid synthesis | 17.58 | 348.33 | 102.47 | 28115.10 |
| GLPKS4 | GLAREA10034 | Pneumocandin B_0_ biosynthesis |  |  | 305.70 | 26.43 |
| GLNRPS4 | GLAREA10035 | Pneumocandin B_0_ biosynthesis |  |  | 127.86 | 31.88 |
| GLligase | GLAREA10043 | Pneumocandin B_0_ biosynthesis |  |  | 104.76 | 18.52 |
| GLHYD | GLAREA10032 | Pneumocandin B_0_ biosynthesis |  |  | 161.74 | 35.20 |
| GLOXY1 | GLAREA10033 | Pneumocandin B_0_ biosynthesis |  |  | 351.33 | 112.41 |
| GLOXY2 | GLAREA10041 | Pneumocandin B_0_ biosynthesis |  |  | 263.52 | 60.70 |
| GLP450-1 | GLAREA10030 | Pneumocandin B_0_ biosynthesis |  |  | 138.89 | 47.96 |
| GLP450-2 | GLAREA10031 | Pneumocandin B_0_ biosynthesis |  |  | 235.26 | 58.57 |
